# The Instrument Set for Generating Fast Adiabatic Passage

**DOI:** 10.1007/s00723-012-0372-3

**Published:** 2012-07-13

**Authors:** T. Czechowski, M. Baranowski, A. Woźniak-Braszak, K. Jurga, J. Jurga, P. Kędzia

**Affiliations:** 1 Laboratory of EPR Tomography, Institute of Materials Technology, Faculty of Mechanical Engineering and Management, Poznan University of Technology, Piotrowo 3A St., 60-965 Poznan, Poland; 2 High Pressure Physics Division, Department of Physics, Adam Mickiewicz University, Umultowska 85, St., 61-614 Poznan, Poland

## Abstract

The design and construction of a high-performance, low-cost, and easy to assemble adiabatic extension set for homebuilt and commercial spectrometers is described. Described apparatus set was designed for the fast adiabatic passage generation and is based on direct digital synthesizer DDS. This solution gives generator high signal to noise ratio, phase stability even during frequency change which is only possible in expansive commercial high-end hardware. Critical synchronization and timing issues are considered and solutions are discussed. Different experimental conditions and techniques for the measurements are briefly discussed. The proposed system is very flexible and might be used for the measurement of low-frequency nuclear magnetic resonance.

## Introduction

Fast adiabatic passage is a fusion of continuous-wave (CW) and pulse techniques. It has significant advantages. During an experiment we are able to rotate all spins by the same angle at the same time without having a high-homogeneity *B*_1_ magnetic field. It is a very important feature in nuclear magnetic resonance (NMR) and electron paramagnetic resonance (EPR) imaging spectrometers because it considerably decreased unwanted artifact into signal intensities which normally have to be corrected [1]. The stability of the external magnetic field *B*_0_ during the spectroscopy measurement is not restricted (in reasonable limits—3 dB probe band) because during the fast adiabatic passage we always find a resonance condition. Amplifier power may be significantly limited but it needs to fulfill an adiabatic passage condition described by Eq. (3).

Only a few papers from the past describe apparatus for the fast adiabatic passages creation. For example, a field sweep of a triangular form was described in Refs. [2–4]. Powles [5] and Parker [6] introduced a field sweep of a trapezoidal form. The modification of the commercial Varian HA-100 NMR spectrometer to permit frequency sweep adiabatic fast passage measurements of T_1_ was described in Ref. [7]. This spectrometer was operated in the lock mode, making signal averaging possible. Those techniques were based on electronic solutions available in the early 1970s.

The adiabatic fast passage (AFP) method requires relatively simple and low-cost additional equipment which can be used with almost any commercial or homebuilt NMR spectrometers. The purpose of this work was to describe the versatile experimental setup, apparatus for this purpose and procedure used in our laboratory for the AFP experiments, and to discuss briefly this method for measuring relaxation times and NMR and the possibility to perform a new EPR (radio frequency) *T*_1_ imaging.

Fast adiabatic passage could be useful even in EPR imaging which has a significant limitation of using pulse techniques caused by short relaxations times, and long acquisition time for CW techniques, especially for oximetry. An increase in speed was achieved using a direct detection of resonances without a low-frequency modulation that would allow arbitrary rapid scan of the field [8–14]. Instead of the second modulation, the method consists of rapid sweep of the magnetic field (at a frequency of 1–10 kHz) or rapid frequency scan [15], which can be performed in the sinusoidal or triangular pattern. The lack of the second modulation eliminates the need for phase detection, which makes it possible to obtain an absorption spectrum, not its first derivative. Consequently, the measurement time of a single projection can be, respectively, reduced to as low as 100 μs for rapid scan of the magnetic field and 15 μs for frequency scan. In practice, spectrum accumulation is required due to the low signal-to-noise (S/N) ratio. Despite that, the rapid scan technique has been successfully supplanting the traditional CW method due to multiple reduction of measurement time. The rapid scan technique is very useful to determine *T*_2_ radical relaxation times, and by speed up sweep rate, shorter *T*_2_ relaxation times could be detected. Recently, by used rates of up to 60 MG/s, the *T*_2_ relaxation times for a stabile organic BDPA radical were obtained at the X-band [16]. Even though in the rapid scan technique the time between consecutive transitions through resonances is shorter than that for fast adiabatic passage, the fast adiabatic passage method needs a shorter measurement time for three-dimensional oxygenation imaging. It is because for oxygen concentration EPR imaging by rapid scan method a time consuming spectral–spatial image reconstruction technique have to be performed, which is not needed for the fast adiabatic passage imaging method [1]. Despite that an unwanted background signal that is the result of interaction between the scanning field, the external magnetic field, and the resonator occurs in the rapid scan methods, makes signal analysis more problematic. In addition, the hardware needed for rapid scan methods by magnetic field sweep [13] are more demanding than for fast adiabatic methods, which makes the fast adiabatic method easier to perform. It should be noted that there are extremely short spin–spin *T*_2_ and spin–lattice *T*_1_ relaxation times for most of free radical electrons. That is why the most frequently used pulse EPR diagnostic is transversal component of magnetization detection technique which is used for *T*_1_ and *T*_2_ relaxation times detection. However, significant resonance broad lines connected with short relaxations times render this method difficult and in lots of cases useless for EPR imaging. The free induction decay signal time shortening in gradient presence is an additional problem. It makes FID detection impossible because the signal appears in the dead time of the receiver.

A new technique of the spin–lattice *T*_1_ relaxation time measurement based on the fast adiabatic passage and on the longitudinal component of the magnetization [1] is devoid of the aforementioned limitations.

**Fig. 1:**
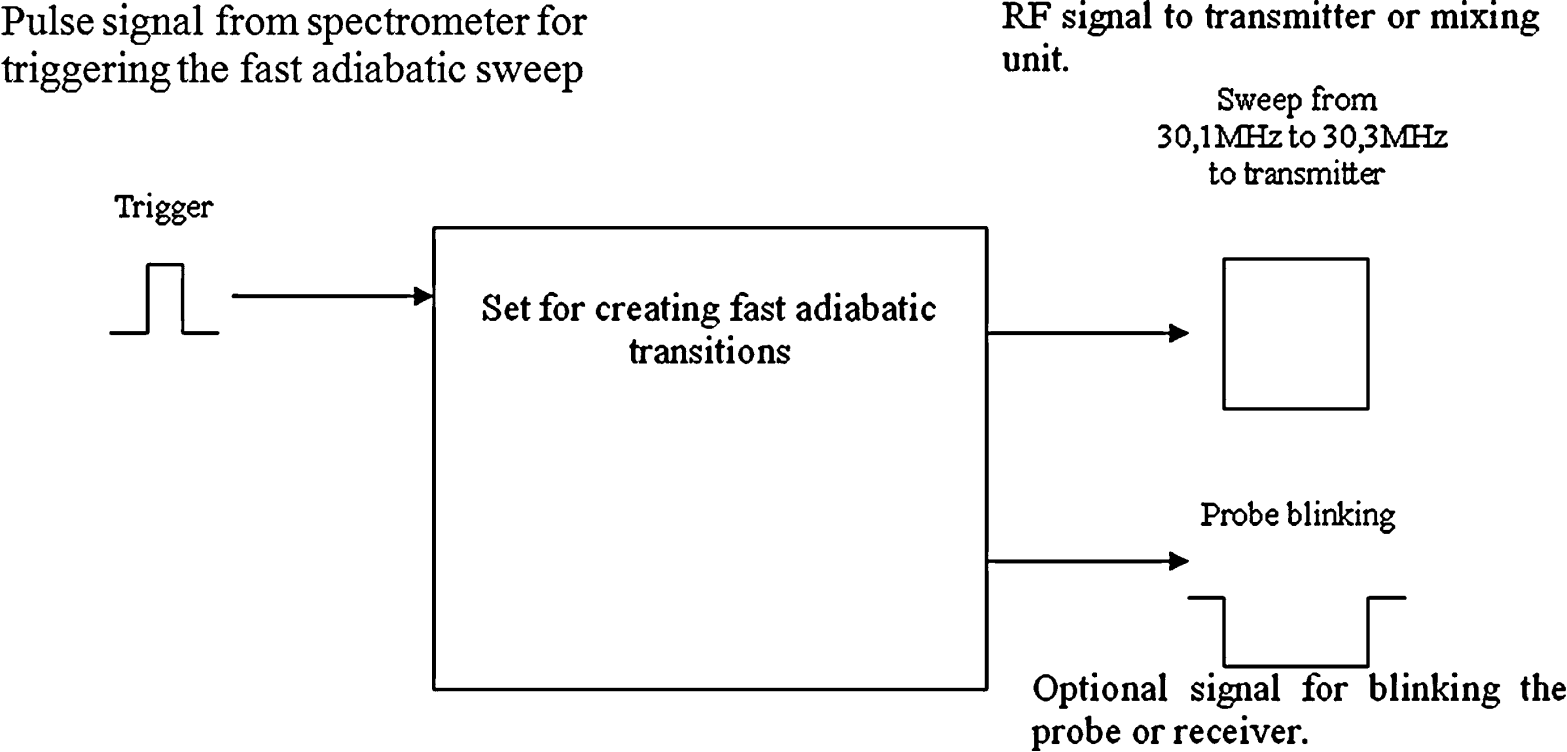
Block diagram of the set for creating fast adiabatic passages

Proposed in this work, the basic equipment necessary for the AFP experiments is a *B*_1_ magnetic field sweep generator based on direct digital synthesis (DDS) and RISC microcontroller. The block diagram of the set is shown in Fig. 1. The DDS technique emerged in the early 1980s and had been probably known in principle for several years before that. In those days the output frequency was limited to a few MHz by the available logic and digital-to-analogue converters’ (DACs) speed. Recently, due to advancement in semiconductor technology, the attainable performance has increased by leaps and bounds. Now DDS chips are made up of gallium arsenide and may provide direct output frequencies, even 500 MHz output frequency. DDS is a very good solution especially for NMR carrier generator. It provides a remarkable frequency resolution and allows the direct implementation of frequency, phase, and amplitude modulation. Detailed information about DDS is available online in http://www.analog.com. Only a few papers describe the application of DDS for the NMR purpose. According to Chinese scientists, it is possible to construct almost a perfect receiver based on digital quadrature detection using the DDS technique [17]. This method requires only one receiver channel. It eliminates phase and matching errors between channels in a standard solution. Other examples of applications of DDS in radiospectroscopy were described in Refs. [18–21].

However, DDS requires serial or parallel programming from an output source, for example, a microcontroller. For the idea of this work ATmega32 was chosen for this purpose. It is a powerful CMOS 8-bit microcontroller based on the AVR-enhanced RISC architecture. By executing instructions in a single-clock cycle, the ATmega32 achieves throughputs approaching 1 MIPS per megahertz allowing optimizing power consumption versus processing speed.

The authors carefully studied devices available on the market and decided to choose an AD9851 180 MHz direct digital synthesizer produced by Analog Devices. The AD9851 is a highly integrated device that uses an advanced DDS technology, coupled with an internal high-speed, high-performance D/A converter. When connected to an accurate clock source, the AD9851 generates a stable frequency and phase-programmable digitized analog output sine wave. This sine wave can be used directly as a frequency source; however, for NMR purpose an external amplifier with input and output impedance designed at 50Ω must be added.

## Detailed Description of the Apparatus Set

**Fig. 2:**
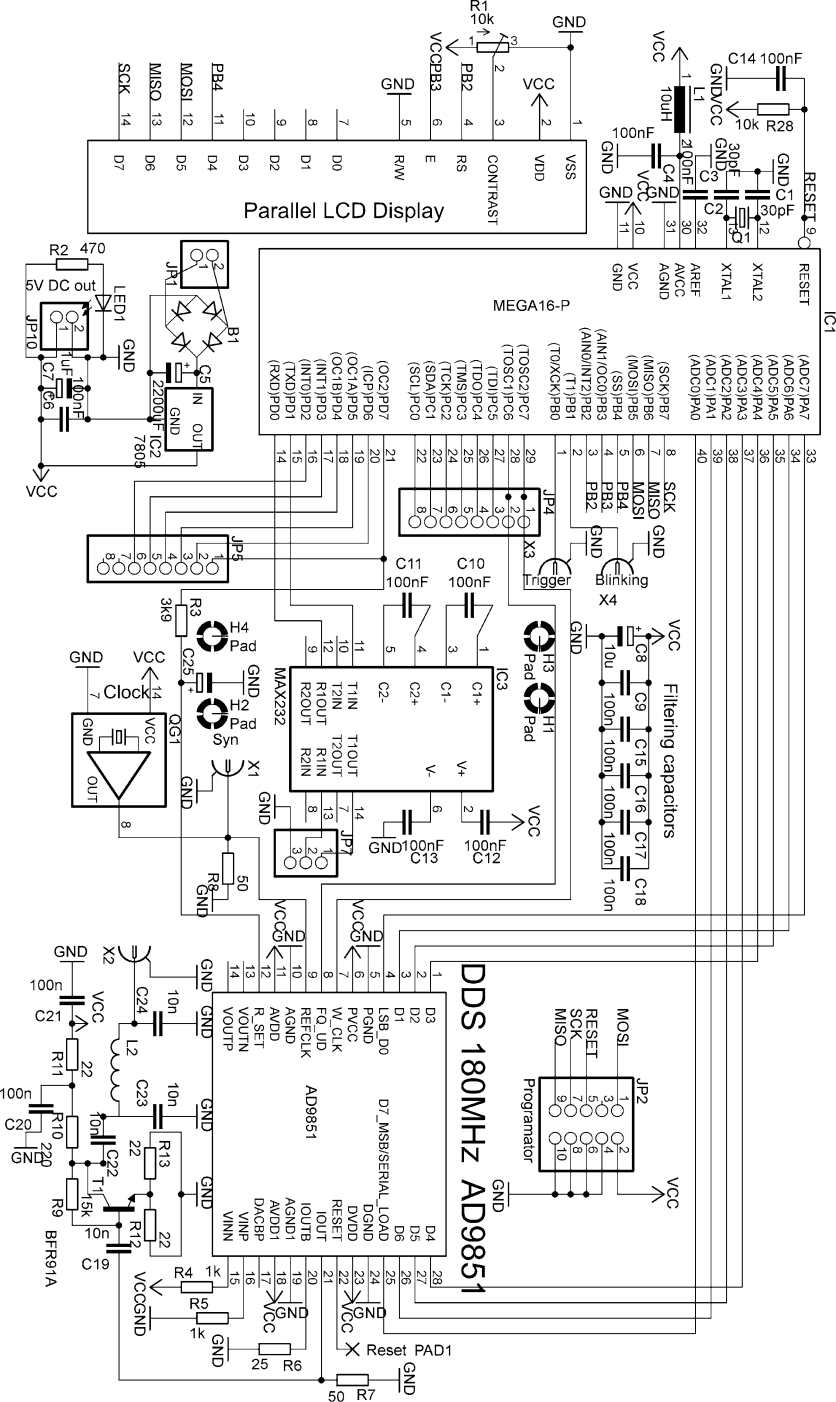
Detailed scheme of the apparatus set for creating fast adiabatic transitions

Figure 2 shows a detailed schema of the constructed apparatus. Phase and frequency synchronization is possible using X1 input/output. If used as an input, a QG1 generator does not have to be mounted on the board and an external synchronization signal must be applied from the spectrometers.

An external trigger signal applied to the X3 input starts a program for the generation frequency ramp by DDS.

Because of timing, the frequency ramp must be written in the internal memory of the microcontroller and therefore an external communication port rs232 was added. To adjust logic levels between the TTL and rs232 standards a MAX232 was added to the circuit. This solution provides the possibility of the configuration of all parameters directly from PC computer.

The output amplitude of carrier frequency might be regulated using pulse with modulation PWM form microcontroller output PD7 connected to a low-pass filter R3 C25. At the output of this filter connected to R_SET DDS the input amplitude is proportional to the PWM duty cycle.

All set parameters may be permanently controlled on an LCD 24 × 4 character display.

The fast adiabatic passage generation system is a prototype; thus there was added a possibility for in-system programming to make a modification of the processor program using a JP2 connector based on AVR standard.

The output amplifier was based on a BFR91A low-power and high-frequency silicon transistor and the amplification was designed at 20 dB in a common emitter configuration.

At the end of the power amplifier a simple low-pass filter C23, C24, L2 was added. The above-described configuration is engineered for maximum amplitude, phase, and frequency stability. To apply this system in spectrometers which work at the frequency beyond 80 MHz, a frequency multiplier must be used.

## Test Experiment

All tests and experiments were carried out on a homebuilt NMR pulse spectrometer which operates at 30.2 MHz. This spectrometer contains a unique double-coil high-homogeneity *B*_1_ probe designed for off-resonance experiments. A detailed construction of the probe was described in Ref. [22]. The spectrometer allows also performing the measurement of relaxation times in the laboratory frame at lover *B*_1_ homogenity for testing fast adiabatic passage. For testing the apparatus set two kinds of experiments were performed. In one of them, water was used as a sample and in the second one a sample was solid-state TMAI (tetramethylammonium iodide). The samples were placed in a glass tube 1 cm long and 0.6 cm in diameter.

Three different methods of measurements of the spin–lattice relaxations times *T*_1_ in the laboratory frame were applied:

1. the inversion recovery method,
2. the saturation method
3. the fast adiabatic passage.

All measurements were carried out at the same temperature of 303 K.

In the first experiment, a pulse sequence [π – *t* − π/2] was used to obtain the spin–lattice relaxation time *T*_1_. The first π RF pulse rotates the magnetization *M*_0_ into the negative plane. After the inversion time *t*, which allows relaxing the magnetization along the +*z* axes, the second π/2 pulse is applied, and then the free induction decay (FID) signal is observed whose amplitude *M*_z_ depends on the time *t*. The time *T*_1_ was determined by fitting the magnetization curve to the equation:

1 $$ M_{\text{z}} \left( t \right) = M_{0} \left( {1 - 2\exp \left( { - \frac{t}{{T_{1} }}} \right)} \right). $$

**Fig. 3:**
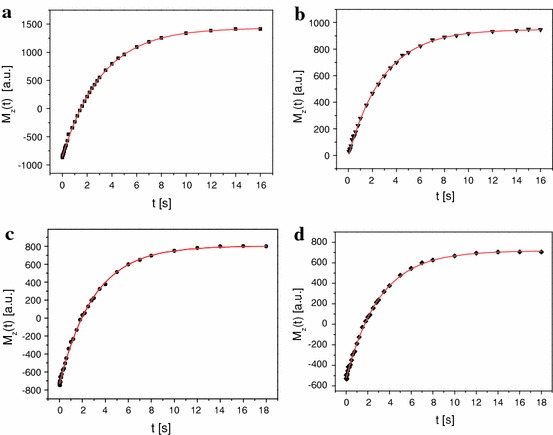
Recovery of the longitudinal magnetization *M*_z_ as a function of *t* for water: **a** the inversion recovery method, **b** the saturation method, **c** the adiabatic passage. *B*_1_ = 10 G, **d** the adiabatic passage *B*_1_ = 20 G, respectively

Figure 3a presents the recovery of the longitudinal magnetization *M*_z_ with the fitting curve obtained by the inversion recovery method.

In the second experiment, the spin–lattice relaxation time T_1_ was obtained using a saturation pulse sequence [n × π/2 – *t* − π/2]. The series of RF π/2 pulses at an interval of several hundred microseconds causes the saturation of the magnetization. Next, the longitudinal magnetization *M*_z_ begins to recover and after time *t* the second π/2 pulse is applied, and the FID signal is observed. The magnitude of the longitudinal magnetization *M*_z_ as a function of *t* was fitted to a function:

2 $$ M_{\text{z}} \left( t \right) = M_{0} \left( {1 - \exp \left( { - \frac{t}{{T_{1} }}} \right)} \right), $$

and presented in Fig. 3b.

The third experiment consisted in the complete inversion of magnetization by the adiabatic fast passage which was characterized by the following conditions: the time to sweep through the linewidth should be, on the one hand, much longer than the period of precession in the magnetic field *B*_1_. On the other hand, it should be much shorter than relaxation times *T*_1_ and *T*_2_:

3 $$ \frac{2\pi }{{\gamma_{p} B_{1} }} \ll \frac{{2B_{1} }}{{\frac{{{\text{d}}H_{0} }}{{{\text{d}}t}}}} \ll T_{1} ,T_{2}, $$

where *B*_1_ is the applied RF magnetic field in gauss, d*H*/d*t* is the sweep rate in gauss/second which could be performed either by magnetic field or frequency sweep methods, and γ_*p*_ is the proton gyromagnetic ratio in rad/s gauss. In Eq. (3) it is assumed that *T*_1_ is equal to *T*_2_, which is true for liquids. However, the condition of fast adiabatic passage for solids must take into account a local field *H*_L_ [23–27]:

4 $$ \frac{1}{{\gamma_{p} B_{1} }} \ll  \frac{{H_{\text{L}} }}{{\frac{{{\text{d}}H_{0} }}{{{\text{d}}t}}}} \ll T_{1} . $$

For our experimental assumptions

γ_*p*_ = 26752 rad/Gs; *B*_1_ = 10 G; d*t* = 1 ms, *f*_0_ = 30.2 MHz, and the swept rate of the magnetic field amounting to

5 $$ \frac{{{\text{d}}H}}{{{\text{d}}t}} = \frac{{{\text{d}}\omega }}{{\gamma_{p} {\text{d}}t}} = \frac{{2\pi \times 0.2 \times 10^{6} \,1/{\text{s}}}}{{26752\,{\text{rad}}/{\text{G}}\,{\text{s}} \times 1 \times 10^{ - 3} {\text{s}}}} = \frac{1256637}{26.7} = 47 \times 10^{3} \,{\text{G}}/{\text{s}}, $$

was performed by frequency swept methods, and the time to sweep through the linewidth is equal to

6 $$ \frac{{2B_{1} }}{{\frac{{{\text{d}}\omega }}{{\gamma_{p} {\text{d}}t}}}} = \frac{{2 \times 10\;{\text{G}}}}{{47 \times 10^{3} \;{\text{G}}/{\text{s}}}} = 425 \times 10^{ - 6} \;{\text{s}}, $$

and the period of precession

7 $$ \frac{2\pi }{{\gamma_{p} B_{1} }} = \frac{2\pi }{{26752 \times 10\,{\text{G}} \times {\text{rad}}/{\text{G}}\,{\text{s}}}} = 2.3 \times 10^{ - 5} \;{\text{s}}. $$

The calculated adiabatic condition in our experiment

$$ 23 \times 10^{ - 6} \ll 425 \times 10^{ - 6} \ll T_{1} ,T_{2} $$

and a similar one for *B*_1_ = 20 G

$$ 12 \times 10^{ - 6} \ll  851 \times 10^{ - 6} \ll T_{1} ,T_{2} $$

are well satisfied.

**Fig. 4:**
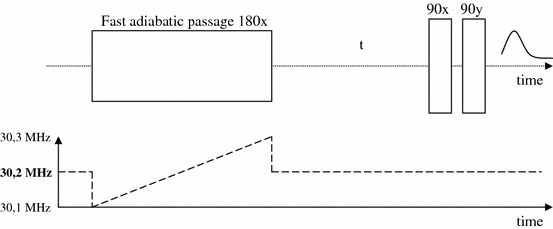
Fast adiabatic passage sequence and the ramp shape

The fast adiabatic passage sequence and the ramp shape applied for testing the apparatus set are shown in Fig. 4.

Figure 3c, d illustrates the dependence of the longitudinal magnetization *M*_z_ as a function of *t* in the fast adiabatic passage for *B*_1_ = 10 G and *B*_1_ = 20 G, respectively. The calculated relaxation time *T*_1_ according to Eq. (1) are collected in Table 1.

**Table 1:** Values of the spin–lattice relaxation times *T*_1_ obtained by different methods

Sample	The inversion recovery method T1 (s)	The saturation method T1 (s)	The adiabatic passage *B*_1_ = 10 G T1 (s)	The adiabatic passage *B*_1_ = 20 G T1 (s)
Water	3.1	2.9	3.0	3.1
TMAI	15.6	14.7	15.7	16.4

**Fig. 5:**
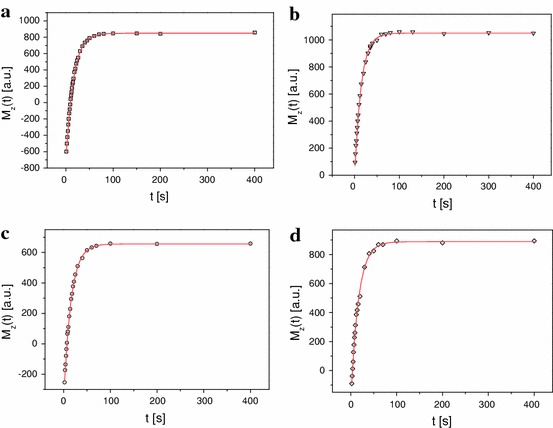
Recovery of the longitudinal magnetization *M*_z_ as a function of t for TMAI: **a** the inversion recovery method, **b** the saturation method, **c** the adiabatic passage *B*_1_ = 10 G, **d** the adiabatic passage *B*_1_ = 20 G, respectively

Similar experiments were performed for solid-state tetramethylammonium iodide (TMAI) (Fig. 5). The obtained results of the relaxation times *T*_1_ are posted in Table 1, which shows the values of spin–lattice relaxation times obtained using different methods. It follows that for the research liquid the relaxation times *T*_1_ obtained by the adiabatic passage are longer compared with those obtained by the saturation method because in the adiabatic passage experiment heterogeneity *B*_1_ magnetic field are neglected. The differences among the relaxation times *T*_1_ received for the solid TMAI are clear with the longest relaxation times measured by the adiabatic passage both *B*_1_ = 10 G and *B*_1_ = 20 G.

## Conclusions

All experiments performed for the purpose of this paper show that the projected and made extension set for the commercial and homebuilt spectrometers works fine. Fast adiabatic passage could be very useful in numerous types of experiments where high homogeneity of *B*_1_ is needed and the power of transmitter is limited. This gives the possibility of applying the AFP technique especially in low-frequency NMR. The microcontroller application provides several advantages such as speed, flexible programming to fit the experimental conditions, and almost unlimited expandability. While DDS gets a high signal-to-noise ratio, phase stability even during a frequency change and output frequency could be simply multiplied.

## References

[1] K. Jurga, M. Baranowski, T. Czechowski, J. Jurga, E. Szczesniak, patent application **P390887** (2010), **EP11001983** (2011)

[2] Nederbragt GW, Reilly CA (1956). J. Chem. Phys..

[3] Pritchard AM, Richards RE (1966). Trans. Faraday Soc..

[4] Atkins PW, Loewenstein A, Margalit Y (1969). Mol. Phys..

[5] Powles JG, Bunsenges Ber (1963). Z. Physik. Chem..

[6] Parker RG, Jonas J (1970). Rev. Sci. Instrum..

[7] Chien M, Samulski ET, Wade CG (1972). Rev. Sci. Instrum..

[8] Joshi JP, Ballard JR, Rinard JA, Quine RW, Eaton SS, Eaton RR (2005). J. Magn. Reson..

[9] Stoner JW, Szymański D, Eaton SS (2004). J. Magn. Reson..

[10] Tsetlin M, Dhami A, Eaton SS, Eaton GR (2007). J. Magn. Reson..

[11] Tsetlin M, Czechowski T, Eaton SS, Eaton GR (2008). J. Magn. Reson..

[12] Tsetlin M, Czechowski T, Quine RW, Eaton SS, Eaton GR (2009). J. Magn. Reson..

[13] Quine RW, Czechowski T, Eaton GR (2009). Magn. Res. Eng..

[14] Tsetlin M, Rinard GA (2011). J. Magn. Reson..

[15] Tsetlin M, Rinard GA (2011). J. Magn. Reson..

[16] Mitchell DG, Quine RW, Tsetlin M, Weber RT, Meyer V, Avery A, Eaton SS, Eaton GR (2011). J. Phys. Chem. B.

[17] Li GY, Xie HB (1999). Rev. Sci. Instrum..

[18] Y. Nakashima, M. Fukuda, K. Matsuta, T. Miyake, M. Mihara, K. Minamisono, T. Sumikama, M. Tanigaki, T. Minamisono, *OULNS Annual Report*, (2001), pp. 60–63

[19] Stolbunov RN, Chichikov SA, Lundin AG (2005). Instrum. Exp. Tech..

[20] R.E. Meller, D.L. Hartill, *Proc. Particle Accelerator Conf. 2339* (2003). doi:10.1109/PAC.2003.1289111

[21] Begus S, Fefer D (2007). Meas. Sci. Technol..

[22] Baranowski M, Woźniak-Braszak A, Jurga K (2011). J. Magn. Reson..

[23] Janzen WR (1971). J. Chem. Phys..

[24] Jansen WR (1973). J. Magn. Reson..

[25] Jansen WR (1973). J. Magn. Reson..

[26] Jansen WR, Cyr TJR, Dunell BA (1968). J. Chem. Phys..

[27] Abragam A, Bleaney B (1970). Electron paramagnetic resonance of transition ions.

